# Supplementary material for: De novo mutations in the GTP/GDP-binding region of RALA, a RAS-like small GTPase, cause intellectual disability and developmental delay
Source: PLoS Genet. 2018 Nov 30;14(11):e1007671. doi: 10.1371/journal.pgen.1007671 (PMC6291162; doi:10.1371/journal.pgen.1007671)
Supplement: S1 Text — (PDF) [file pgen.1007671.s001.pdf]

Appendix S1: Supplemental Materials and Methods

Genome/exome sequencing and variant analysis

Site A

Genome sequencing (GS) was performed on proband 1 and both parents in a research setting at HudsonAlpha. For proband 11, only the proband was sequenced by WGS. In both cases, variant filtering and prioritization were performed as previously described [1]. Sanger validation was performed in a CAP/CLIA-certified laboratory.

Site B

For proband 2, exome sequencing (ES) of the proband and both parents was performed in a research setting at Charles University. Exome capture was performed using the SeqCap EZ Human Exome Library v3.0 (Roche NimbleGen, WI). ES was performed on the Illumina HiSeq 1500 platform (Illumina) according to the manufacturer's recommendations for paired-end 101 bp reads. The ES reads were mapped to the hg19 reference genome using Novoalign (Novocraft Technologies, Selangor, Malaysia, http://www.novocraft.com/products/novoalign/, Accessed 11 July 2016). SAM to BAM conversion and PCR duplicate removal were performed using Picard (Broad Institute of MIT and Harvard, Cambridge, MA, USA, http://broadinstitute.github.io/picard/, Accessed 11 July 2016). Genome Analysis Toolkit (GATK) [2] was used for local realignment around indels, base recalibration, variant recalibration and genotyping. Variants were annotated using the GEMINI framework [3] and subjected to several levels of filtering. Common variants (allele frequency >0.01) from public human genetic variation databases (dbSNP, 1000 Genomes, Exome Variant Server (EVS), and the Exome Aggregation Consortium (ExAC), the Genome Aggregation Consortium (gnomAD)) or from the in-house database were removed. Filtering was also performed according to the predicted severity of the impact of the variants, inheritance patterns, presence in in-house candidate gene lists and phenotype match with published mutation carriers.

Site C

ES was performed on proband 3 plus parents in a clinical setting at Ambry Genetics using the SeqCapEZ VCR 2.0 (Roche NimbleGen) and sequenced on the HiSeq 2000 Sequencer (Illumina, San Diego, CA, USA) and analyzed as previously described [4, 5].

Site D

Probands 4 and 5 are monozygyotic twins. ES (Roche SeqCap MedExome/Illumina NextSeq 500 2x150bp high output) was performed on proband 4 and both parents in a research setting at La Pitié-Salpêtrière Hospital, Paris. Alignment and variant calling were performed using standard software (bwa -0.7.12, samtools-1.1, picard-tools-1.121, GenomeAnalysisTK-2014.3-17-g0583018, including Haplotype caller, FastQC 0.10.1). Variants were annotated using SNPEff-4.2 and dbNSFP. Variants were filtered to require 1) minor allele frequency in ExAC <1% and 2) impact on the coding sequence (missense, stop gained, stop loss, start loss, frameshift and inframe indel, splice donor and acceptor variants). Sanger confirmation was performed in all four family members

using Thermo Fischer Big Dye Terminator V3 and Applied Biosystem 3730 sequencer with POP7. Sequences were analyzed with Applied Biosystem Seqscape v2.5.

Site E

Trio ES was done on proband 6 and his parents after written informed consent was obtained through an institutional review board-approved research study at the Institute for Genomic Medicine at Columbia University (protocol AAAO8410). DNA was extracted from maternal, paternal, and proband samples, exome sequenced on a HiSeq 2500 with the Kapa Biosystem's Library Preparation Kit, and exome captured with Nimblegen SeqCap EZ v.3.0. Trio sequence data were analyzed with an updated version of our established trio sequencing framework [6] which identifies ‘‘qualifying’’ genotypes not observed in the parents, 4,435 control individuals from the Institute for Genomic Medicine, or two external databases of 6,503 and 60,706 control individuals provided by the National Heart, Lung, and Blood Institute (NHLBI) Exome Sequencing Project (ESP6500SI, March 2013 release) and the Exome Aggregation Consortium (ExAC Browser v.0.3, January 2015 release), respectively.

Site F

For probands 7, 8, 9, and 10, using genomic DNA from the proband plus parents, the exonic regions and flanking splice junctions of the genome were captured using either the Clinical Research Exome kit (probands 7, 8 and 9, Agilent Technologies, Santa Clara, CA) or the IDT xGen Exome Research Panel v1.0. (Proband 10, Integrated DNA Technologies, Inc., Skokie, IL). Sequencing was performed on an Illumina system with 100 bp or greater paired-end reads. Reads were aligned to human genome build GRCh37/UCSC hg19, and analyzed for sequence variants using a custom-developed analysis tool. Additional sequencing technology and variant interpretation protocol has been previously described [7]. The general assertion criteria for variant classification are publicly available on the GeneDx ClinVar submission page (http://www.ncbi.nlm.nih.gov/clinvar/submitters/26957/).

Data used in calculation of observed frequency of variation in *RALA*.

Proband	Site	Site Name	Number of DD/ID-affected probands sequenced as trios at site
1	A	HudsonAlpha Institute for Biotechnology	400
2	B	Charles University	54[a]
3	C	Ambry Genetics	2763
4	D	La Pitié-Salpêtrière Hospital	514
5	D	La Pitié-Salpêtrière Hospital	NA[b]
6	E	Institute for Genomic Medicine at Columbia University Medical Center	650
7, 8, 9, 10	F	GeneDx	11759
11	A	HudsonAlpha Institute for Biotechnology	NA[c]

[a]Proband 2 was not included in calculations due to small cohort size.
[b]Proband 5 was not included in calculations as he represents a monozygotic twin of Proband 4.
[c]Proband 11 was not included in calculations as only the proband was sequenced, and we could not discern inheritance of the *RALA* variant in this proband.

Supplemental References

1. Bowling KM, Thompson ML, Amaral MD, Finnila CR, Hiatt SM, Engel KL, et al. Genomic diagnosis for children with intellectual disability and/or developmental delay. Genome Med. 2017;9(1):43.
2. McKenna A, Hanna M, Banks E, Sivachenko A, Cibulskis K, Kernytsky A, et al. The Genome Analysis Toolkit: a MapReduce framework for analyzing next-generation DNA sequencing data. Genome Res. 2010;20(9):1297-303.
3. Paila U, Chapman BA, Kirchner R, Quinlan AR. GEMINI: integrative exploration of genetic variation and genome annotations. PLoS Comput Biol. 2013;9(7):e1003153.
4. Farwell Hagman KD, Shinde DN, Mroske C, Smith E, Radtke K, Shahmirzadi L, et al. Candidate-gene criteria for clinical reporting: diagnostic exome sequencing identifies altered candidate genes among 8% of patients with undiagnosed diseases. Genet Med. 2017;19(2):224-35.
5. Farwell KD, Shahmirzadi L, El-Khechen D, Powis Z, Chao EC, Tippin Davis B, et al. Enhanced utility of family-centered diagnostic exome sequencing with inheritance model-based analysis: results from 500 unselected families with undiagnosed genetic conditions. Genet Med. 2015;17(7):578-86.
6. Zhu X, Petrovski S, Xie P, Ruzzo EK, Lu YF, McSweeney KM, et al. Whole-exome sequencing in undiagnosed genetic diseases: interpreting 119 trios. Genet Med. 2015;17(10):774-81.

7. Tanaka AJ, Cho MT, Millan F, Juusola J, Retterer K, Joshi C, et al. Mutations in SPATA5 Are Associated with Microcephaly, Intellectual Disability, Seizures, and Hearing Loss. Am J Hum Genet. 2015;97(3):457-64.
