# Supplementary material for: De novo mutations in the GTP/GDP-binding region of RALA, a RAS-like small GTPase, cause intellectual disability and developmental delay
Source: PLoS Genet. 2018 Nov 30;14(11):e1007671. doi: 10.1371/journal.pgen.1007671 (PMC6291162; doi:10.1371/journal.pgen.1007671)
Supplement: S2 Text — (PDF) [file pgen.1007671.s002.pdf]

S2 Text. Clinical Summaries

Patient 1 is a 12 year old female with a history of developmental delay, autism and complex partial epilepsy. She had slow language development and first walked at 2 years of age. She is reported to have relative microcephaly, slanted palpebral fissures, slightly flat nasal bridge and has some subcanthal folds. She has a history of migraines with a component of vomiting, excessive drooling, and continued issues with balance. Seizures present as "staring off" episodes for three to four seconds. Mother and maternal uncle of the proband are reported to have learning disabilities and maternal grandmother has Crohn's disease, autoimmune issues, and a history of migraines. Physicians report a normal brain MRI and an abnormal EEG with multifocal spikes identified. The proband was 48.3 cm (30%ile) and 3374 g (46%ile) at birth. At last examination (11y), she was 134.6 cm (10%ile) and 27.22 kg (4%ile).

Proband 2 is a male, born from the first pregnancy of healthy unrelated parents. The age of the mother and father were 37 and 57 years, respectively. The father had one healthy son from his previous marriage. The family had no history of mental or behavioral impairment. The boy was born in the 41st week of gestation by Caesarean section due to prolonged labor and abnormal fetal monitoring findings. The birth weight was 3500 g (50%ile), length was 49 cm (25%ile) and Apgar score was 10-10-10. Clifford syndrome was apparent after birth. The boy needed special early postnatal care including parenteral feeding. He also suffered from neonatal jaundice treated with phototherapy lasting 30 hours. Later on he showed hypotonia, had feeding problems, and failure to thrive. Developmental delay has been evident since infancy with lack of movements, lack of interest in toys, and a limited contact with his parents.

The boy was able to sit with support at 18 months of age. He produced first sounds at six months of age, but full speech has never developed as he only produces syllables. EEG examination at 11 months showed no abnormality. Brain MRI identified a small periventricular gliosis, possibly resulting from a perinatal hypoxic ischemic insult. Psychological examination revealed severe to profound intellectual disability with severe speech impairment and stereotypic and autoaggressive behaviors.

At 20 months the boy showed a gracile habitus with macrocephaly; his weight was 83 cm (3-10%ile), height was 10.4 kg (25%ile) and head circumference was 51 cm (90-97%ile). He exhibited broad prominent forehead, hypertelorism, epicanthal folds, depressed nasal bridge, broad nasal tip and short columella, full cheeks, deep philtrum, wide mouth, exaggerated Cupid's bow, low-set ears with a slight dorsal rotation and bright curly hair. His lower extremities were thin and short with overlapping fingers.

At 30 months his relative macrocephaly was even more pronounced and he showed a broad forehead, high hairline, slight frontal bossing and depressed nasal bridge. He could stand with support on a wide basis and moved only on all fours and showed signs of the paleocerebellar syndrome with hypotonia. He was diagnosed with autism spectrum disorder. His psychomotor development corresponded to a 10-month-old child. Brain MRI showed persisting periventricular gliosis, incomplete myelination (although improved compared with the previous examination) and dysplastic (shorter and wider) corpus callosum.

This proband has a VUS in *FLNA*, NM_001456.3:c.1816G>C, (p.V606L). The variant is hemizygous in proband, and was inherited from a heterozygous unaffected mother but not from the unaffected grandfather.

Proband 3 is a 7 year old male born from the third pregnancy of healthy unrelated parents. Positive family history related to patient's phenotype: Maternal half uncle of the proband has autism. Ethnicity is reported as Filipino, Hawaiian, Chinese, Caucasian (countries unspecified), consanguinity is unknown. The patient was born to a G3P1SAb mother at 40.6 weeks gestation, weighing 8 pounds 4.1 oz (64%ile), length of 50.5 cm (55%ile), and OFC of 36.5 cm (64%ile). Apgars were 8 and 9. Mother reported decreased fetal movements compared to first pregnancy. By 1 year of age, he was noted to have developmental delays, mild hypotonia, and slow weight gain. At 2 years of age, it was noted that he had global developmental delay, asthma, could use a spoon and fork, had a ‘clumsy run’, frequent falls, and was in the process of toilet training. He was also diagnosed with autism by 2 y. At age 5, he measured 15.876 kg (4.57%ile) and 104 cm (5.11%ile). He started saying words at about 6y. At 7y 5m, he measured 18.7 kg (2%ile) and 112.5 cm (1%ile) and has global developmental delay, hypotonia, autism spectrum disorder, and excessive drooling. He is presumed to have intellectual disability although the level of his cognitive development may preclude standardized testing at this point. Maladaptive behaviors include self-injurious behavior and sleep onset issues. He is not very interested in other children, and has a restricted diet. He is reportedly saying some phrases and single words. He can say about 20 words. He is nondysmorphic, has no seizures, hearing and vision are normal.

Brain MRI/ MR spectroscopy was performed at 1y 11m that was reported as normal. An echocardiogram at 7y 5m noted the following: Mitral valve appears thickened and redundant, annulus measures mildly dilated, and there is mild prolapse and trivial mitral regurgitation. The tricuspid valve appears mildly redundant with borderline proplapse. There is trivial tricuspid regurgitation. The aortic valve is trileaflet. The annulus measures mildly dilated. There is borderline proplapse. Array CGH, Fragile X, methylation for PW/AS, seq/del/dup for *MECP2*, and SMA exons 7 and 8 were negative. AutismNext NGS panel reported 3 VUSs: paternally-inherited *POGZ* (p.V1303F, absent from gnomAD) and *RAI1* (p.M1849V, found in gnomAD 24x), and maternally-inherited *SLC6A8* (p.H593R, found in gnomAD in 9 heterozygotes). As the clinician determined these variants did not explain the proband’s phenotype, trio exome sequencing was performed at age 5.

Probands 4 and 5 are monozygotic twin brothers born at 38 weeks gestation to healthy parents with weights of 2,730 g (9%ile) and 2,850 g (13%ile), respectively, OFC 33.5 cm (15%ile) and 34 cm (20%ile), respectively, and a length of 46 cm (6%ile) for both. Disease history is similar in both boys during the first years of life. First concerns arose when they were 6-month-old because of delayed milestones, truncal hypotonia and poor eye contact. The boys never acquired sitting position and never spoke. Patient 4 had a first tonic seizure at 6 years. He received sodium valproate, then levetiracetam + clobazam. He had at most several short seizures per day that were occasionally triggered by fever. His brother had first epileptic absences at 13 years treated with levetiracetam + clobazam.

At last examination at the age of 16.5 years, both boys were profoundly intellectually disabled, wheelchair bound, poorly used their hands and eye contact was still limited. Both had scoliosis, marked truncal hypotonia, gum hypertrophy, long fingers with extensible joints, and epicanthal folds. OFC measurements were 54.5 cm (proband 4) and 55 cm (proband 5). Brain MRI were unremarkable except for thin corpus callosum.

Proband 6 is a preterm (32 wks) product of DCDA twin pregnancy (healthy twin sister) with history of severe global developmental delay, ataxia, failure to thrive, seizure disorder (generalized and myoclonic seizure, with static encephalopathy), undescended testicles and dysmorphic features. He is wheelchair-bound and nonverbal. The proband is able to move his legs to stand with assistance, make some sounds, and sit. He is not able to walk. Also he has some strength in his upper extremities, much more than lower extremities, and attends physical therapy. The proband is G-tube fed in addition to PO feeding. PediaSure, enteral formula and solid food are fed mostly by mouth. Current medications include Valproic acid, Albuterol, Polyethylene Glycol, PediaSure, and Carnitine.

At 13 yrs of age, OFC measured 56cm (90%ile) and weight was approx. 82lbs (10%ile). The 13 year old male was wheelchair-bound, appeared awake, alert, makes sounds, was poorly interactive, and well nourished. He appeared to be in Tanner stage 1-2 and had a myopathic face, mild wheezing and notable drooling. He exhibited axial hypotonia, increased muscle tone on lower extremities with decreased range of motion, strength 1-2/5 (difficult to assess), and was not able to walk. No abnormalities of the nose, ears, oropharynx, neck, cardiovascular system, cranial nerves, or chest were noted. He did exhibit mild down slanting palpebral fissures, long eyelashes (eyes: PERRLA, EOMI), bitemporal narrowing, and a symmetric, large head. A deep tendon reflex noted in the lower extremities (L2-L3), bilaterally.

Karyotype, SNP array, 53 gene epilepsy panel, mtDNA sequencing were all reported to be normal. ES of the proband and mother only in 2013 identified heterozygous variation in *HSOB3*, *KCNQ*, and *ALG2* gene. Subsequent targeted testing in the father revealed that all three variants were inherited from the unaffected father and not reported as causal. An MRI showed abnormalities in myelination and of the corpus callosum, and volume loss.

Proband 7 was born full term (39 weeks gestational age) via normal spontaneous vaginal delivery with a birth weight of 3430 g (50%ile), birth length 50 cm (58%ile), head circumference 34.5 cm (42%ile). Apgar scores were 9 and 10 at 1 and 5 minutes, respectively, and the proband was discharged on the third day of life. Postnatally, she had a history of early colic, irritability and vomiting. The parents mentioned that a milk allergy was diagnosed, but with a special diet she improved. The pediatrician noted hypotonia and developmental delay at 3-4 months of age. She smiled between six and eight months, held her head up at 1.5 years, and rolled over at 12 months. She doesn't sit or walk. The first tooth was at a normal age. The soft spot closed at a normal age.

At 18 months, previously observed dystonia and choreic movements disappeared. Testing for myotonic dystrophy (*DMPK* gene and triplet expansion) was negative, Pompe disease was ruled out, testing for abnormalities of neurotransmitters was negative. Uric acid in

plasma is elevated. Chromosome testing, metabolic screen, and EEG were normal. MRI was normal initially though later a hypophysial cyst was identified. Electromyogram shows some repetitive myotonic or pseudomyotonic complexes in the muscles that were tested. However, muscular biopsy showed no specific changes.

Developmental delay, intellectual disability with learning disability and speech delay, involuntary movements, sensorial issues, increased tolerance to pain and hypotonia have been reported. The parents also report that she has some tendency to protrude the tongue, has increased salivation, and hemangiomas.

On physical examination at 2y 8m, her height was 92 cm (51%ile), weight was 10.78 kg (3%ile), and head circumference measured 48.5 cm (56%ile). She exhibits central hypotonia with developmental delay, no speech, a wide prominent forehead, minor synophrys, epicanthal folds, short philtrum with prominent pillars, and a small somewhat triangular mouth. No abnormal ocular movement was observed, which was reported in the past. She has a minor depression in the midline of the chest, a very small supernumerary nipple on the left side of the chest, a questionable increase in prepubic fat, a minor nevus in the left lower leg, and hemangiomas involving the occipital area, the back, over the midthoracic spine and the sacrococcygeal area. In the lumbosacral area, there is also some remnant of mongolian spot. She has some minor ridge dysplasia on both soles. There are zygodactylous triradii involving the second, third, and fourth toes and clinodactyly of the fifth toes. Finger pads of the hands are slightly conic, proximal and distal axial triradius with an S-pattern in the left hypothenar area, and proximal and intermediate in the right palm. There exist two minor bridges producing some tendency to simian crease transitional 1. Mainline endings were symmetrical, being A5', B7, C9, D11. The right finger patterns were all ulnar loops, however, on the left fingers she has a whorl, an apparently tented arch, U, then a whorl that is certainly a central pocket radial loop in the fourth finger, which is unusual, and in the fifth finger also an ulnar loop. Parents did not show the same features, however the father exhibits syndactyly between the second and third toes.

In summary, the proband has central hypotonia with delayed developmental milestones, features suggestive of myotonia or paramyotonia congenita, very tiny polythelia, and more striking hemangiomas. There is some ridge dysplasia, conic finger pads and minor findings on dermatoglyphics.

This proband has a *de novo* variant in *SHANK2*, NM_012309.3:c.3301G>A, (p.A1101T).

Proband 8 was born by cesarean section and did well until 3 to 4 months of life, when he was noted to have developmental delay. At age 4 months, he had myoclonic seizures and was treated with Zonegran. Initial MRI of the brain at that time showed a structural brain malformation with dysplastic and thin corpus callosum, and areas of polymicrogyria in the right frontal lobe anterior to the central sulcus and over the right parietal head region.

Proband 8 was last examined at 3.5 years of age. He has been off Zonegran for 6 to 7 months and continues seizure free. His mood is good and he typically sleeps well. He has been healthy and review of systems is normal for other body systems. He attends a Cerebral Palsy preschool and receives physical, occupational and speech therapy. He has vision therapy as well, wears glasses, and is doing alternate eye patching. He also is undergoing hippotherapy that he enjoys and works well with. The mother notes he has made some slow developmental

progress. He has almost achieved complete head control, but is unable to sit without assistance.

At 3.5 years old, height was 86 cm (<2%ile), weight was 11.8 kg (<2%ile), and OFC was 51 cm (75%ile). His vital signs were stable and he was afebrile. General examination shows relative macrocephaly. He is in diapers. His anterior fontanel was closed. His general peripheral vascular examination shows no swelling, edema or redness. Neurologic examination showed he is alert and his eyes were open. He was quiet during examination. He would smile briefly for the mother, but would not consistently track. Cranial nerves 2-12 showed his pupils were equal and react to light directly and consensually and his disks are sharp. He has a full range of extraocular movements. Formal visual fields and hearing were not assessed. His face is symmetric. His tongue is midline without fasciculations and his gag was positive. Motor examination shows no definite hand preference. He has a decrease in axial tone and a slight increase in tone in his periphery with decreased bulk and decreased strength. His deep tendon reflexes are 2 plus and 3 plus and symmetric in the upper and lower extremities and both plantar responses are extensor. No abnormal movements were seen. When placed in the sitting position, he has approximately 75% to 90% head control and sits with his back slightly straighter, but still with a C-curve. He appears to have slightly increased tone in the left compared to the right side. I can elevate his left foot to 5 degrees above neutral and the right 10 to 15 degrees above neutral. He has an alternating esotropia noted x2. He will roll in both directions, but will not bear weight when held in standing and cannot get to sitting on his own.

On the date of this visit he had an electroencephalogram performed that captured awake, drowsiness, and sleep, and he had photic stimulation performed. There were no epileptiform discharges and he had diffuse slowing of the background rhythm. A recent MRI showed polymicrogyria over the right greater than left supraventricular frontal and parietal lobes as well as bilateral insulin and supratemporal gyri. He had hypoplastic appearance in the splenium and an asymmetric, prominent linear orientation of the right central sulcus.

This proband has a heterozygous variant in *SCN1A*, NM_006920.4:c.560G>C, (p.R187Q). It was inherited from a heterozygous unaffected father. According to the referring clinician, the observed phenotypes are not consistent with Dravet syndrome.

Proband 9 had an uncomplicated pregnancy and was delivered vaginally at 38 weeks gestation with birth weight of 7 lbs 8 oz (40%ile) and length of 19 in (24%ile). He has had no major health issues. Mild sleep apnea was improved with removal of his tonsils and adenoids. Head MRI at 28 months of age found mildly hypoplastic corpus callosum and cerebellar vermis at the lower limits of normal in size. Vision and hearing evaluations have not found any significant abnormalities. SNP array, fragile X testing, CK, CBC, CMP and TSH were all normal. ES found a *de novo* variant in *RALA*.

From a developmental standpoint, he was not walking until 2 years of age. Speech has been delayed and at 3 years of age he only had 1-2 words used appropriately. At 4-1/2 years of age, he has a small number of 3 words sentences used appropriately. He has mild fine motor delays. He is social and interactive. He is the 2nd child for his parents. His older brother is healthy with normal development. There is no pertinent family history.

Physical exam at 3 years 9 months of age found a height of 100.2 cm (46%ile), weight of 15.3 kg (40%ile). His head circumference measured in the 75-80%ile. He had mild

dolichocephaly with a tall forehead and bitemporal narrowing. He had a mildly flat midface with residual epicanthal folds. Palate was high arched. He had mild generalized hypotonia.

Proband 10 was born by scheduled C-section at 39 weeks gestations age, and was the product of an unremarkable pregnancy. His birth weight was 4.28 kg (93%ile), birth length was 53.3 cm (88%ile), and head circumference was 39 cm (97%ile). There were no issues reported in the neonatal period, with the exception of mild hypoglycemia. This patient was initially assessed at 6 months of age due to a history of abnormal brain MRI findings, and an increase in head circumference velocity, which was first noted at 4 months of age. At 6 months of age, he had difficulties in supporting his head. He was unable to fix and follow consistently.

At age of 2 years, this patient has global developmental delay and macrocephaly. He is able to lift his head and chest off the floor when placed in prone position. He is able to roll over from back to front and vice versa. He is not able to sit or crawl. He is able to stand with significant support. The patient makes vocalizations, and has one word. He is responds to commands mainly by facial expressions and sometimes by turning his head to view named people and objects. He does not yet point. He is orally fed. There are no known choking episodes. He has no known history of seizures. Ophthalmologic exam reveals cycloplegic refraction is +1.50 in both eyes, and is otherwise normal. There is likely cortical vision impairment. The patient has an older healthy brother who also has a large head size. Both of his parents are healthy and well. His mom's head circumference is 56 cm, and his father's head circumference is 61.6 cm. On extensive family history, there are some individuals with large head size on the paternal side of the family. There is no known family history of developmental delay or seizures. Patient is of French Canadian, English, Polish and Ukrainian descent. There is no known consanguinity in his family.

On physical exam at 2 years of age, this patient displays significant macrocephaly with head circumference >98%ile. There was no evidence of cleft palate, but he does have a high arched palate. He has tall and broad forehead with bitemporal narrowing. There was also a deep nasal bridge and tented upper lip. The ears were normally formed but low placed. There was no preauricular pits or tags. The spine examination was unremarkable with no kyphosis, scoliosis or sacral dimple noted. Cardiovascular exam was normal. Abdomen was soft with no evidence of organomegaly. Neurological exam revealed the presence of a significantly decreased central tone and increased peripheral tone. His hands were predominantly fisted, although at times with open palms. There was a brisk deep tendon reflexes with no evidence of clonus.

MRI showed multiple areas of polymicrogyria involving both cerebral hemispheres predominantly seen in the rolandic and perisylvian region bilaterally however also extending into the anterior aspect of frontal lobes and superior aspect of temporal lobes. Tiny cysts are observed in the hypoechoic bilaterally. There are focal areas of parenchymal thinning, especially in the peritrigonal/periventricular region bilaterally, worse on the left. There is mild asymmetry in the myelination of the periventricular white matter, more accentuated on the left corticospinal tract when compared to the right. The corpus callosum is diffusely thin however the posterior aspect is significantly thin compared to the anterior segments. Suspected agenesis or severe hypoplasia of the splenium and rostrum. The

configuration of the supratentorial ventricles is abnormal probably secondary to the arrangement of the white matter tracts which is likely abnormal.

The patient was found to have a 1.349 Mb duplication at chromosome 1q21.1-q21.2. The duplication was found to be paternally inherited. While patient does have some phenotypic features of this duplication, including macrocephaly and developmental delay, the physician's impression was that patient's brain MRI findings could not be explained by the chromosome 1q21.1-q21.2 microduplication.

Proband 11 was born at 35 weeks gestation, via vaginal delivery with breech presentation with resulting hypoxic ischemic encephalopathy (APGAR scores of 1,2,3, and 4 at 1, 5, 10 and 15 minutes, respectively). Measurements at birth were length of 45.7 cm (5%ile) and weight of 1.96 kg (1%ile). He spent 66 days in the neonatal intensive care unit where his hospital course was complicated by Grade III and Grade IV intraventricular hemorrhages at day 10 of life, bronchopulmonary dysplasia, and poor oral feeding requiring G-tube placement.

At birth he was noted to have ambiguous genitalia with a small genital tubercle (0.5cm) with an inferior urethral opening, fused labioscrotal folds and no palpable gonads. Pelvic ultrasound identified no uterus, ovarian, or testicular structures. Endocrinological evaluation revealed low testosterone, undetectable anti-Mullerian hormone, and karyotype consistent with 46,XY. Additional genetic testing included a 15-gene 46-XY Disorder of Sexual Development commercial gene panel (including *DHCR7*), which was normal. Dysmorphological exam showed a long face with hypotonic appearance, widely-spaced eyes, upslanting palpebral fissures, a high-arched palate with a central groove (not cleft), and retrognathia.

At 5 months of age, he had a supraglottoplasty and at 6 months of age he contracted Respiratory Syncytial Virus and experienced respiratory failure necessitating tracheostomy. He developed seizures at 9 months of age, EEGs demonstrated multifocal epileptogenicity, and he was not responsive to phenobarbital, oxcarbazepine, clobazam, levetiracetam, or a ketogenic diet. Over time his baseline EEG worsened, and he experienced decreased level of alertness and interactions. At 16 months of age, he was 73 cm long (1%ile), 9.8 kg (9%ile), and had an OFC of 44 cm (<1%ile). He is profoundly hypotonic and globally developmentally delayed at 2 years, 1 month.

Brain MRI as a neonate demonstrated cystic encephalomalacia of the posterior left frontal and anterior left parietal lobes, focal leukomalacia of the left frontal lobe, and two small areas of subcortical leukomalacia of the right frontal lobe. At 17 months of age, MRI showed bilateral white matter cerebral volume loss, cortical atrophy in the left parietal lobe, and cerebellar atrophy, along with hemosiderin the dependent ventricles bilaterally.

Array comparative genomic hybridization + SNP array demonstrated no clinically relevant copy number changes, but did show 24 large regions of homozygosity encompassing 27% of the genome, indicating a first-degree relationship between the parents. Proband-only GS was performed and a heterozygous nonsense variant in *RALA* (c.526C>T; p.R176X) was indicated. No homozygous variants of clinical suspicion were identified. Mitochondrial genome sequencing was negative.
