# Supplementary material for: De novo mutations in the GTP/GDP-binding region of RALA, a RAS-like small GTPase, cause intellectual disability and developmental delay
Source: PLoS Genet. 2018 Nov 30;14(11):e1007671. doi: 10.1371/journal.pgen.1007671 (PMC6291162; doi:10.1371/journal.pgen.1007671)
Supplement: S1 Table — (PDF) [file pgen.1007671.s003.pdf]

S1 Table. Sequencing sites, experiment types, and data used in calculation of observed frequency of variation in *RALA*.

Proband	Site	Site Name	Experiment Type, Subjects	Research/Clinical	Number of DD/ID-affected probands sequenced as trios at site
1	A	HudsonAlpha Institute for Biotechnology	GS, Trio	Research	400
2	B	Charles University	ES, Trio	Research	54[a]
3	C	Ambry Genetics	ES, Trio	Clinical	2763
4	D	La Pitié-Salpêtrière Hospital	ES, Trio	Clinical	514
5	D	La Pitié-Salpêtrière Hospital	Sanger only, monozygotic twin of proband 4	Clinical	NA[b]
6	E	Institute for Genomic Medicine at Columbia University Medical Center	ES, Trio	Research	650
7, 8, 9, 10	F	GeneDx	ES, Trio	Clinical	11759
11	A	HudsonAlpha Institute for Biotechnology	GS, Proband Only	Research	NA[c]

ES, Exome sequencing; GS, genome sequencing.
[a]Proband 2 was not included in calculations due to small cohort size.
[b]Proband 5 was not included in calculations as he represents a monozygotic twin of Proband 4.
[c]Proband 11 was not included in calculations as only the proband was sequenced, and we could not discern inheritance of the *RALA* variant in this proband.
