# Supplementary material for: De novo mutations in the GTP/GDP-binding region of RALA, a RAS-like small GTPase, cause intellectual disability and developmental delay
Source: PLoS Genet. 2018 Nov 30;14(11):e1007671. doi: 10.1371/journal.pgen.1007671 (PMC6291162; doi:10.1371/journal.pgen.1007671)
Supplement: S2 Table — (PDF) [file pgen.1007671.s004.pdf]

S2 Table. Missense variants present in the gnomAD and Bravo databases. PASS only missense variants from gnomAD (http://gnomad.broadinstitute.org/gene/ENSG00000006451, Accessed June 21, 2018) and Bravo (https://bravo.sph.umich.edu/freeze5/hg38/gene/ENSG00000006451, Accessed June 21, 2018) are included. Combined, 34 unique amino acid residues are affected by variation in these databases. None of these variants lie in the GTP/GDP-binding region. Although data from Bravo were first downloaded based on hg38 (and Freeze 5), reference coordinates shown here represent hg19. Source db, Source database; chr, chromosome; pos, position; ref, reference; alt, alternate.

Source db	chr:pos	ref/alt	Protein Consequence	Allele Count	Allele Number	Allele Frequency
Bravo	7:39726270	G/A	p.Ala2Thr	101	125568	8.04E-04
gnomAD	7:39726270	G/A	p.Ala2Thr	551	277182	0.001988
gnomAD	7:39726309	C/T	p.His15Tyr	1	246252	4.06E-06
gnomAD	7:39726313	A/G	p.Lys16Arg	1	246252	4.06E-06
gnomAD	7:39726321	A/G	p.Met19Val	1	246246	4.06E-06
gnomAD	7:39726375	G/A	p.Asp37Asn	2	245528	8.15E-06
gnomAD	7:39730035	C/A	p.Leu57Ile	1	246174	4.06E-06
Bravo	7:39730058	C/G	p.Ile64Met	1	125568	7.96E-06
gnomAD	7:39730092	G/A	p.Ala76Thr	1	246196	4.06E-06
gnomAD	7:39730108	A/G	p.Asn81Ser	1	246138	4.06E-06
Bravo	7:39730138	G/A	p.Cys91Tyr	1	125568	7.96E-06
Bravo	7:39730159	T/G	p.Met98Arg	1	125568	7.96E-06
gnomAD	7:39730159	T/G	p.Met98Arg	1	244604	4.09E-06
Bravo	7:39730168	T/G	p.Phe101Cys	1	125568	7.96E-06
Bravo	7:39730176	A/G	p.Thr104Ala	1	125568	7.96E-06
gnomAD	7:39730176	A/G	p.Thr104Ala	1	243670	4.10E-06
Bravo	7:39730177	C/T	p.Thr104Ile	1	125568	7.96E-06

Bravo	7:39730179	G/A	p.Ala105Thr	1	125568	7.96E-06
gnomAD	7:39730179	G/A	p.Ala105Thr	7	243106	2.88E-05
gnomAD	7:39736309	G/A	p.Asp117Asn	1	241996	4.13E-06
gnomAD	7:39736314	G/T	p.Glu118Asp	1	30978	3.23E-05
gnomAD	7:39736321	C/G	p.Pro121Ala	2	273986	7.30E-06
gnomAD	7:39736368	G/C	p.Gln136His	1	30972	3.23E-05
gnomAD	7:39736369	G/A	p.Val137Ile	1	246078	4.06E-06
gnomAD	7:39736375	G/C	p.Val139Leu	1	246060	4.06E-06
Bravo	7:39736376	T/C	p.Val139Ala	2	125568	1.59E-05
gnomAD	7:39736376	T/C	p.Val139Ala	2	277014	7.22E-06
gnomAD	7:39736391	A/T	p.Asn144Ile	1	246104	4.06E-06
Bravo	7:39736420	G/T	p.Val154Leu	1	125568	7.96E-06
Bravo	7:39737011	G/T	p.Gly162Val	1	125568	7.96E-06
gnomAD	7:39737011	G/T	p.Gly162Val	2	129724	1.54E-05
gnomAD	7:39737013	C/A	p.Pro163Thr	1	129734	7.71E-06
gnomAD	7:39737017	C/G	p.Ser164Cys	1	129728	7.71E-06
gnomAD	7:39745722	G/A	p.Val167Ile	3	234748	1.28E-05
Bravo	7:39745749	C/G	p.Arg176Gly	1	125568	7.96E-06
gnomAD	7:39745749	C/G	p.Arg176Gly	1	239464	4.18E-06
gnomAD	7:39745750	G/A	p.Arg176Gln	1	240606	4.16E-06
gnomAD	7:39745753	C/T	p.Ala177Val	3	270572	1.11E-05
gnomAD	7:39745761	A/T	p.Met180Leu	1	243174	4.11E-06
gnomAD	7:39745763	G/A	p.Met180Ile	2	243172	8.23E-06
Bravo	7:39745771	G/A	p.Ser183Asn	1	125568	7.96E-06

Bravo	7:39745793	G/C	p.Lys190Asn	1	125568	7.96E-06
gnomAD	7:39745793	G/C	p.Lys190Asn	2	274884	7.28E-06
gnomAD	7:39745803	A/G	p.Ser194Gly	1	244120	4.10E-06
Bravo	7:39745812	A/C	p.Lys197Gln	2	125568	1.59E-05
gnomAD	7:39745812	A/C	p.Lys197Gln	1	30950	3.23E-05
gnomAD	7:39745816	G/T	p.Arg198Ile	3	243564	1.23E-05
gnomAD	7:39745817	A/T	p.Arg198Ser	1	243246	4.11E-06
