# Supplementary material for: De novo mutations in the GTP/GDP-binding region of RALA, a RAS-like small GTPase, cause intellectual disability and developmental delay
Source: PLoS Genet. 2018 Nov 30;14(11):e1007671. doi: 10.1371/journal.pgen.1007671 (PMC6291162; doi:10.1371/journal.pgen.1007671)
Supplement: S3 Table — (PDF) [file pgen.1007671.s005.pdf]

S3 Table. Evidence for association of variation in RAS proteins with rasopathies, at residues corresponding to GTP/GDP-binding regions. Residues most commonly affected by somatic variation in RAS proteins are highlighted in yellow. Residues affected by variation identified here in RALA are highlighted in red. Overlapping or nearby residues associated with RASopathies are highlighted in blue. These residues (and colors) correspond to those shown in the alignment in S7 Figure.

		Support for RAS-associated disease		
**RALA Residue**	**RAS Residue**	**HRAS**	**KRAS**	**NRAS**
G23	G12	Kerr, et al. 2006[1], Aoki, et al. 2005[2]	Nava, et al. 2007[3]	Altmüller, et al. 2017[4]
G24	G13	Aoki, et al. 2005[2]	NR	Altmüller, et al. 2017[4]
V25	V14	NR	Zenker, et al. 2007[5], Schubbert, et al. 2006[6] and others	NR
K128	K117	Kerr, et al. 2006[1], Denayer, et al. 2008[7]	ClinVar Submission (GeneDx)	NR
D130	D119	NR	NR	NR
S157	S145	NR	NR	NR
A158	A146	Chiu, et al. 2017[8], Gripp et al. 2008[9], Zampino, et al. 2007[10]	ClinVar Submission (Invitae)	NR
K159	K147	NR	Stark et al. 2012[11]	NR

Supplemental References

1. Kerr B, Delrue MA, Sigaudy S, Perveen R, Marche M, Burgelin I, et al. Genotype-phenotype correlation in Costello syndrome: HRAS mutation analysis in 43 cases. J Med Genet. 2006;43(5):401-5.
2. Aoki Y, Niihori T, Kawame H, Kurosawa K, Ohashi H, Tanaka Y, et al. Germline mutations in HRAS proto-oncogene cause Costello syndrome. Nat Genet. 2005;37(10):1038-40.
3. Nava C, Hanna N, Michot C, Pereira S, Pouvreau N, Niihori T, et al. Cardio-facio-cutaneous and Noonan syndromes due to mutations in the RAS/MAPK signalling pathway: genotype-phenotype relationships and overlap with Costello syndrome. J Med Genet. 2007;44(12):763-71.
4. Altmuller F, Lissewski C, Bertola D, Flex E, Stark Z, Spranger S, et al. Genotype and phenotype spectrum of NRAS germline variants. Eur J Hum Genet. 2017;25(7):823-31.
5. Zenker M, Lehmann K, Schulz AL, Barth H, Hansmann D, Koenig R, et al. Expansion of the genotypic and phenotypic spectrum in patients with KRAS germline mutations. J Med Genet. 2007;44(2):131-5.
6. Schubbert S, Zenker M, Rowe SL, Boll S, Klein C, Bollag G, et al. Germline KRAS mutations cause Noonan syndrome. Nat Genet. 2006;38(3):331-6.
7. Denayer E, Parret A, Chmara M, Schubbert S, Vogels A, Devriendt K, et al. Mutation analysis in Costello syndrome: functional and structural characterization of the HRAS p.Lys117Arg mutation. Hum Mutat. 2008;29(2):232-9.
8. Chiu AT, Leung GK, Chu YW, Gripp KW, Chung BH. A novel patient with an attenuated Costello syndrome phenotype due to an HRAS mutation affecting codon 146-Literature review and update. Am J Med Genet A. 2017;173(4):1109-14.
9. Gripp KW, Innes AM, Axelrad ME, Gillan TL, Parboosingh JS, Davies C, et al. Costello syndrome associated with novel germline HRAS mutations: an attenuated phenotype? Am J Med Genet A. 2008;146a(6):683-90.
10. Zampino G, Pantaleoni F, Carta C, Cobellis G, Vasta I, Neri C, et al. Diversity, parental germline origin, and phenotypic spectrum of de novo HRAS missense changes in Costello syndrome. Hum Mutat. 2007;28(3):265-72.
11. Stark Z, Gillessen-Kaesbach G, Ryan MM, Cirstea IC, Gremer L, Ahmadian MR, et al. Two novel germline KRAS mutations: expanding the molecular and clinical phenotype. Clin Genet. 2012;81(6):590-4.
