# Supplementary material for: De novo mutations in the GTP/GDP-binding region of RALA, a RAS-like small GTPase, cause intellectual disability and developmental delay
Source: PLoS Genet. 2018 Nov 30;14(11):e1007671. doi: 10.1371/journal.pgen.1007671 (PMC6291162; doi:10.1371/journal.pgen.1007671)
Supplement: S1 Fig — Detailed view of the wild type V25 residue (A) and its substitutions V25M (B) and V25L (C). (PDF) [file pgen.1007671.s006.pdf]

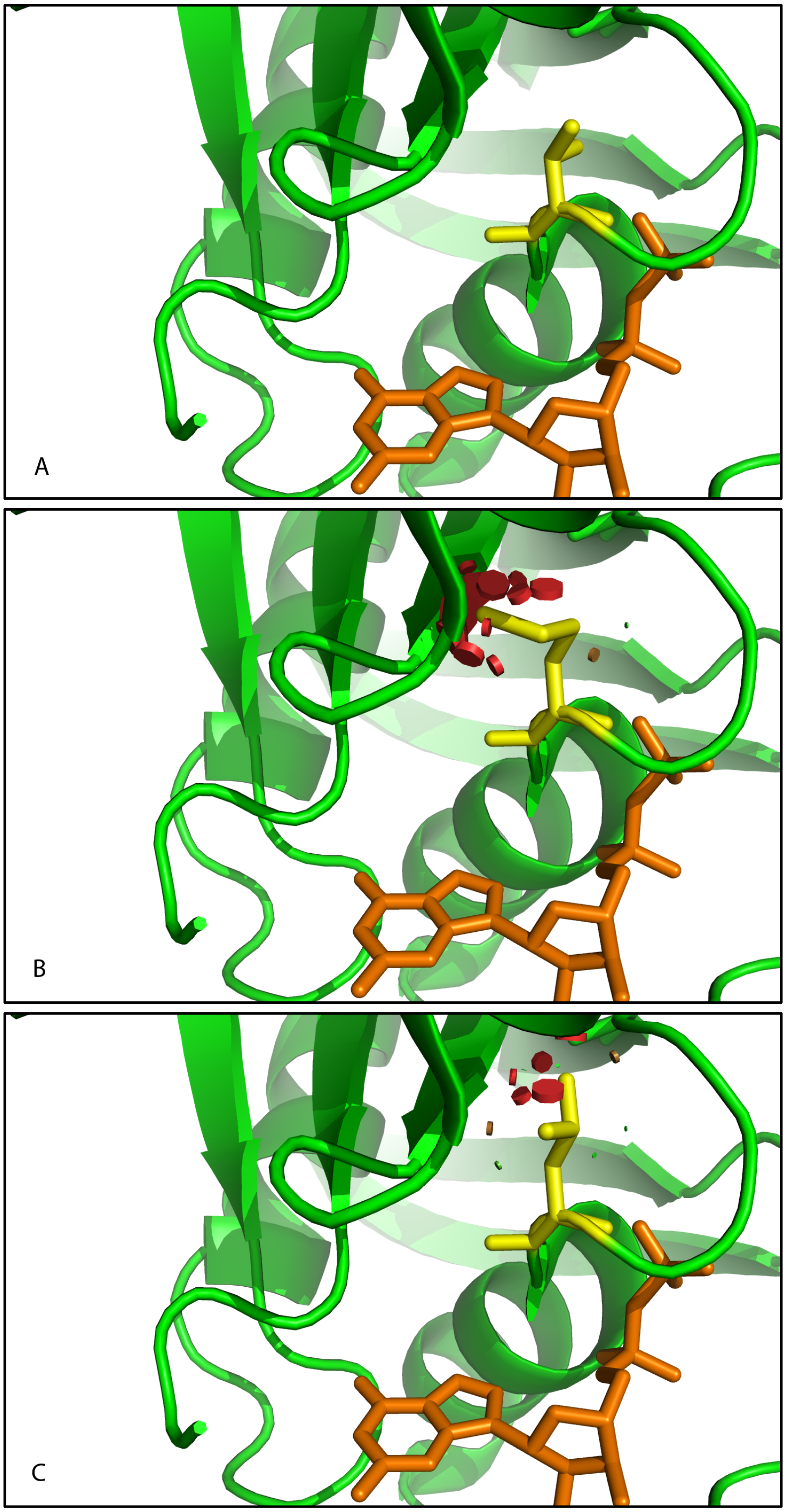

S1 Figure. Detailed view of the wild type V25 residue (A) and its substitutions V25M (B) and V25L (C). Panel A shows the Val25 residue in yellow, while panel B shows substitution of this residue to methionine (in yellow), and panel C the substitution to leucine (in yellow). GDP is shown in orange. Since the new residues are significantly bigger, the Van der Walls diameters of

some of their atoms overlap with diameters of some atoms from the rest of the molecule. These overlaps are shown as red discs with sizes proportional to the size of the overlap. The collisions likely cause a distortion of the structure of the GDP/GTP-binding region.
