# Supplementary material for: De novo mutations in the GTP/GDP-binding region of RALA, a RAS-like small GTPase, cause intellectual disability and developmental delay
Source: PLoS Genet. 2018 Nov 30;14(11):e1007671. doi: 10.1371/journal.pgen.1007671 (PMC6291162; doi:10.1371/journal.pgen.1007671)
Supplement: S2 Fig — (PDF) [file pgen.1007671.s007.pdf]

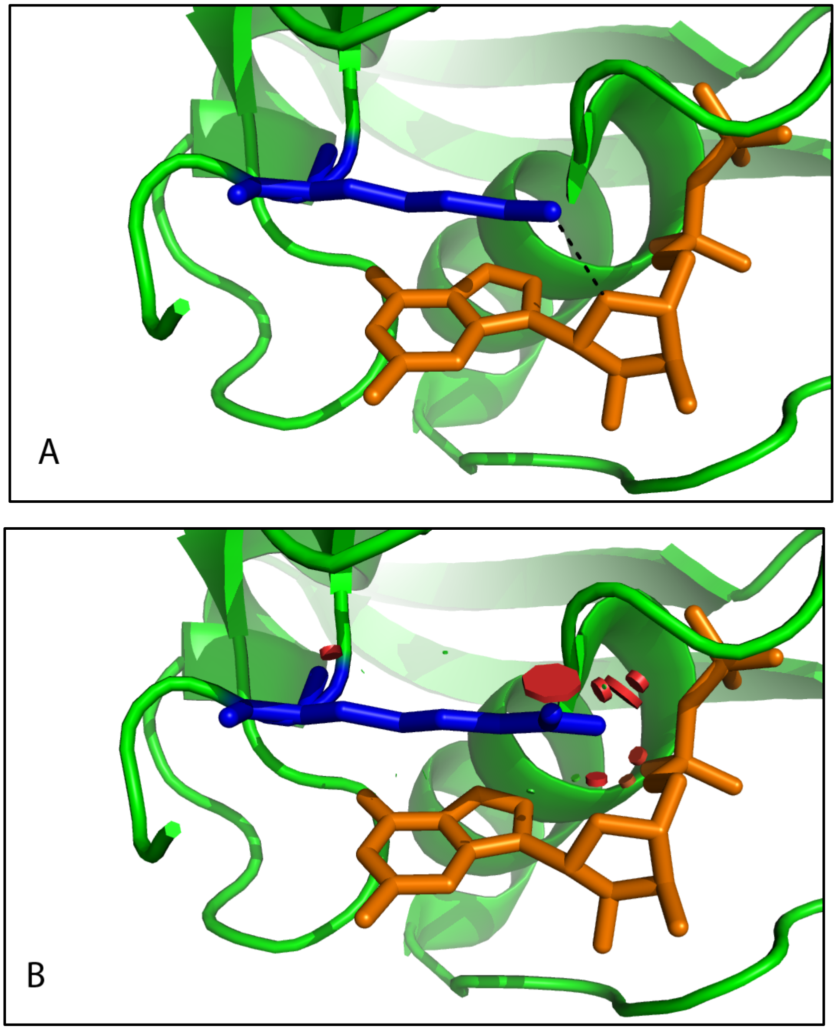

S2 Figure. Detailed view of the wild type K128 residue and its substitution K128R. Panel A shows the lysine residue (blue) with its hydrogen bond with GDP (black dashed line, GDP in orange). Panel B show the substitution for arginine (in blue). The bigger size of the new residue again causes overlaps with other atoms of the protein molecule and a distortion of the GDP/GTP-binding pocket. The overlaps are shown as red discs with sizes proportional to the size of the overlap.
