# Supplementary material for: De novo mutations in the GTP/GDP-binding region of RALA, a RAS-like small GTPase, cause intellectual disability and developmental delay
Source: PLoS Genet. 2018 Nov 30;14(11):e1007671. doi: 10.1371/journal.pgen.1007671 (PMC6291162; doi:10.1371/journal.pgen.1007671)
Supplement: S4 Fig — (PDF) [file pgen.1007671.s009.pdf]

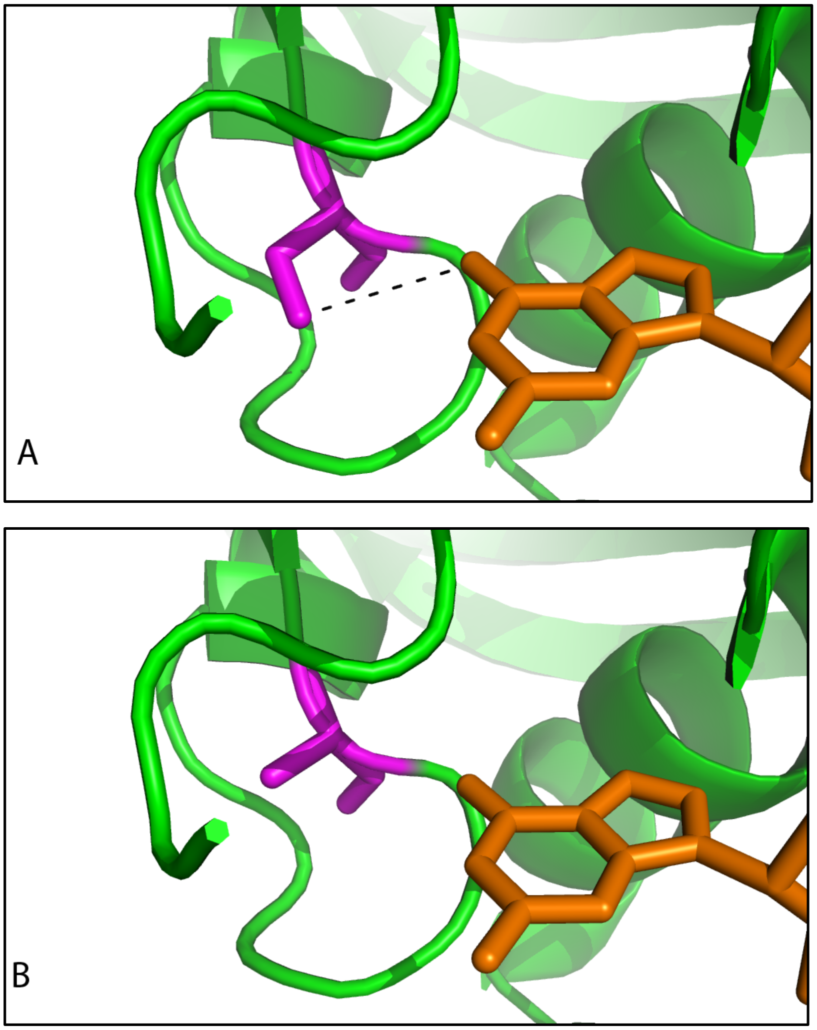

S4 Figure. Detailed view of the wild type S157 residue and its substitution S157A. Panel A shows the serine residue (in magenta) with its hydrogen bond with GDP (black dashed line, GDP in orange). Panel B shows the substitution for alanine (magenta) unable to form the hydrogen bond.
