# Supplementary material for: De novo mutations in the GTP/GDP-binding region of RALA, a RAS-like small GTPase, cause intellectual disability and developmental delay
Source: PLoS Genet. 2018 Nov 30;14(11):e1007671. doi: 10.1371/journal.pgen.1007671 (PMC6291162; doi:10.1371/journal.pgen.1007671)
Supplement: S5 Fig — (PDF) [file pgen.1007671.s010.pdf]

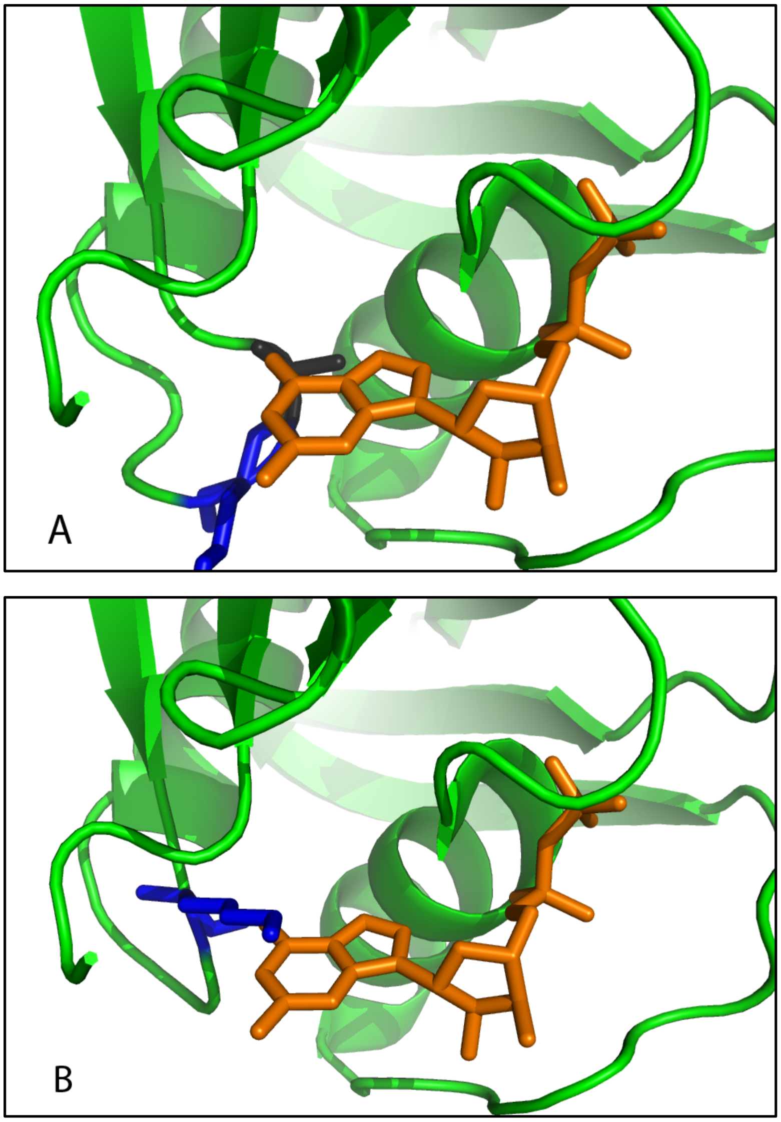

S5 Figure. Detailed view of the wild type A158 residue, neighboring residue K159 and GDP (in orange). Panel A shows the structure with residue A158 present (in black) and panel B shows the deletion of residue A158. Due to the deletion, the residue K159 (blue) is shifted towards the GTP/GDP-binding region and can hinder GTP/GDP binding.
