# Supplementary material for: De novo mutations in the GTP/GDP-binding region of RALA, a RAS-like small GTPase, cause intellectual disability and developmental delay
Source: PLoS Genet. 2018 Nov 30;14(11):e1007671. doi: 10.1371/journal.pgen.1007671 (PMC6291162; doi:10.1371/journal.pgen.1007671)
Supplement: S6 Fig — (PDF) [file pgen.1007671.s011.pdf]

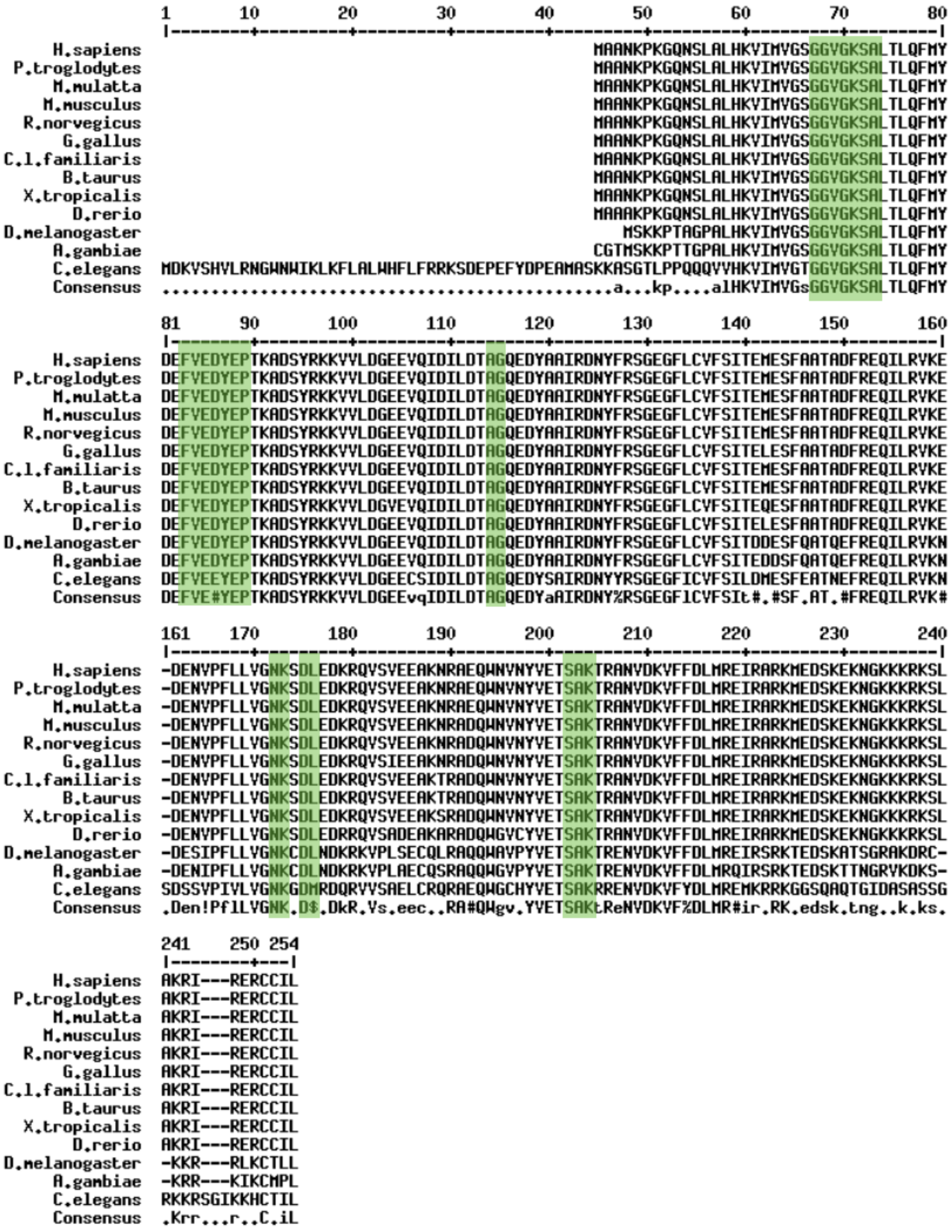

S6 Figure. Alignment of RALA protein sequences across various species. Protein reference sequences of RALA homologs as defined by HomoloGene (NCBI, hid=3942) were aligned using MultAlin (http://multalin.toulouse.inra.fr/multalin/multalin.html). Residues comprising the

GTP/GDP-binding region as defined by computational modeling are highlighted in green. For the Consensus row, uppercase represents a residue conserved across all sequences, lowercase represents a residues conserved across at least half of the sequences, and the following symbols represent one of multiple residues: !, I or V; $, L or M; %, F or Y; #, one of N, B, Q, E, B or Z.
