# Supplementary material for: De novo mutations in the GTP/GDP-binding region of RALA, a RAS-like small GTPase, cause intellectual disability and developmental delay
Source: PLoS Genet. 2018 Nov 30;14(11):e1007671. doi: 10.1371/journal.pgen.1007671 (PMC6291162; doi:10.1371/journal.pgen.1007671)
Supplement: S7 Fig — (PDF) [file pgen.1007671.s012.pdf]

```
                  1                                                  50
RALA_NP_005393.2  MAANKPKGQN SLALHKVIMV GSGGVGKSAL TLQFMYDEFV EDYEPTKADS
HRAS_NP_789765.1  .......... .MTEYKLVVV GAGGVGKSAL TIQLIQNHFV DEYDPTIEDS
KRAS_NP_203524.1  .......... .MTEYKLVVV GAGGVGKSAL TIQLIQNHFV DEYDPTIEDS
NRAS_NP_002515.1  .......... .MTEYKLVVV GAGGVGKSAL TIQLIQNHFV DEYDPTIEDS
       Consensus  .......... .$teyKl!vV GaGGVGKSAL TiQliq#hFV ##Y#PTieDS

                  51                                                100
RALA_NP_005393.2  YRKKVVLDGE EVQIDILDTA GQEDYAAIRD NYFRSGEGFL CVFSITEMES
HRAS_NP_789765.1  YRKQVVIDGE TCLLDILDTA GQEEYSAMRD QYMRTGEGFL CVFAINNTKS
KRAS_NP_203524.1  YRKQVVIDGE TCLLDILDTA GQEEYSAMRD QYMRTGEGFL CVFAINNTKS
NRAS_NP_002515.1  YRKQVVIDGE TCLLDILDTA GQEEYSAMRD QYMRTGEGFL CVFAINNSKS
       Consensus  YRKqVViDGE tcllDILDTA GQE#YsAmRD #YmRtGEGFL CVFaIn#.kS

                  101                                               150
RALA_NP_005393.2  FAATADFREQ ILRVKEDENV PFLLVGNKSD LEDKRQVSVE EAKNRAEQWN
HRAS_NP_789765.1  FEDIHQYREQ IKRVKDSDDV PMVLVGNKCD LA.ARTVESR QAQDLARSYG
KRAS_NP_203524.1  FEDIHHYREQ IKRVKDSEDV PMVLVGNKCD LP.SRTVDTK QAQDLARSYG
NRAS_NP_002515.1  FADINLYREQ IKRVKDSDDV PMVLVGNKCD LP.TRTVDTK QAHELAKSYG
       Consensus  Fadi..%REQ IkRVK#s##V PmvLVGNKcD L...RtV... #A.#lA.syg

                  151                                               200
RALA_NP_005393.2  VNYVETSAKT RANVDKVFFD LMREIRARKM EDSKEKNGKK KRKSLAKRIR
HRAS_NP_789765.1  IPYIETSAKT RQGSRSGSSS SSGTLWDPPG P....M.... ..........
KRAS_NP_203524.1  IPFIETSAKT RQRVEDAFYT LVREIRQYRL K....KI.SK EEKTPGCVKI
NRAS_NP_002515.1  IPFIETSAKT RQGVEDAFYT LVREIRQYRM K....KLNSS DDGTQGCMGL
       Consensus  !p%!ETSAKT Rq.v...f.. l.reir...m .....k...k ..k.......

                  201
RALA_NP_005393.2  ERCCIL
HRAS_NP_789765.1  ......
KRAS_NP_203524.1  KKCIIM
NRAS_NP_002515.1  P.CVVM
       Consensus  ..c.i.
```

**S7 Figure. Alignment of RALA, HRAS, KRAS, and NRAS protein sequences.** Protein reference sequences of RALA, HRAS, KRAS and NRAS were aligned using MultAlin (http://multalin.toulouse.inra.fr/multalin/multalin.html). Residues most commonly affected by somatic variation in RAS proteins are highlighted in yellow. Residues affected by variation identified here in RALA are highlighted in red. Nearby residues associated with RASopathies are highlighted in blue. A green bar is present above residues comprising the GTP/GDP-binding region as defined by computational modeling. For the Consensus row, uppercase represents a residue conserved across all sequences, lowercase represents a residues conserved across at least half of the sequences, and the following symbols represent one of multiple residues: !, I or V; $, L or M; %, F or Y; #, one of N, B, Q, E, B or Z.
