# Supplementary material for: De novo mutations in the GTP/GDP-binding region of RALA, a RAS-like small GTPase, cause intellectual disability and developmental delay
Source: PLoS Genet. 2018 Nov 30;14(11):e1007671. doi: 10.1371/journal.pgen.1007671 (PMC6291162; doi:10.1371/journal.pgen.1007671)
Supplement: S8 Fig — (PDF) [file pgen.1007671.s013.pdf]

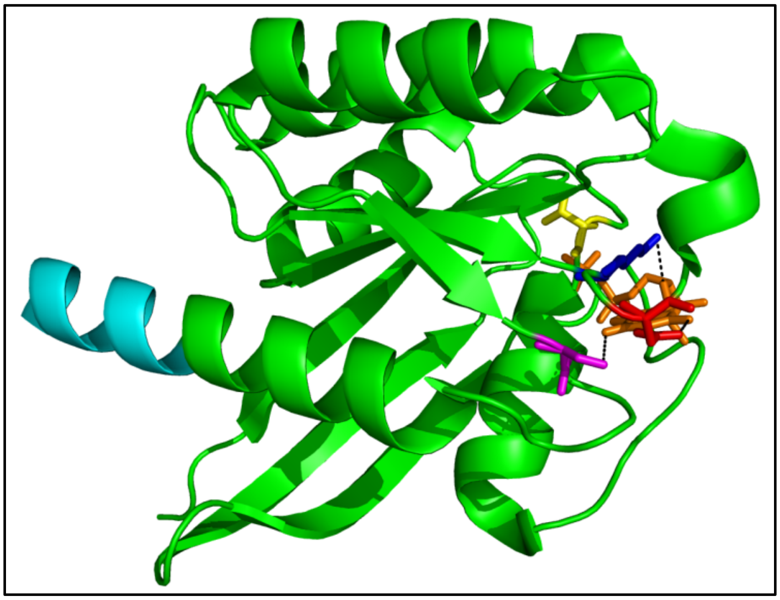

S8 Figure. The extent of the C-terminal truncation (in turquoise) of the RALA molecule caused by the R176* mutation relative to the position of the GDP/GTP-binding region. The RALA protein is shown in a cartoon representation in green, with the C-terminal amino acids 177-206 shown in turquoise. The mutated single-amino acid residues in the GTP/GDP-binding region are shown in licorice representation. V25 is in yellow, K128 in blue, D130 in red, S157 in magenta, and A158 in black. GDP is shown in a licorice representation in orange. Hydrogen bonds between the side chains of these amino acids and GDP are shown as black dashed lines.
