# Supplementary material for: De novo mutations in the GTP/GDP-binding region of RALA, a RAS-like small GTPase, cause intellectual disability and developmental delay
Source: PLoS Genet. 2018 Nov 30;14(11):e1007671. doi: 10.1371/journal.pgen.1007671 (PMC6291162; doi:10.1371/journal.pgen.1007671)
Supplement: S9 Fig — (PDF) [file pgen.1007671.s014.pdf]

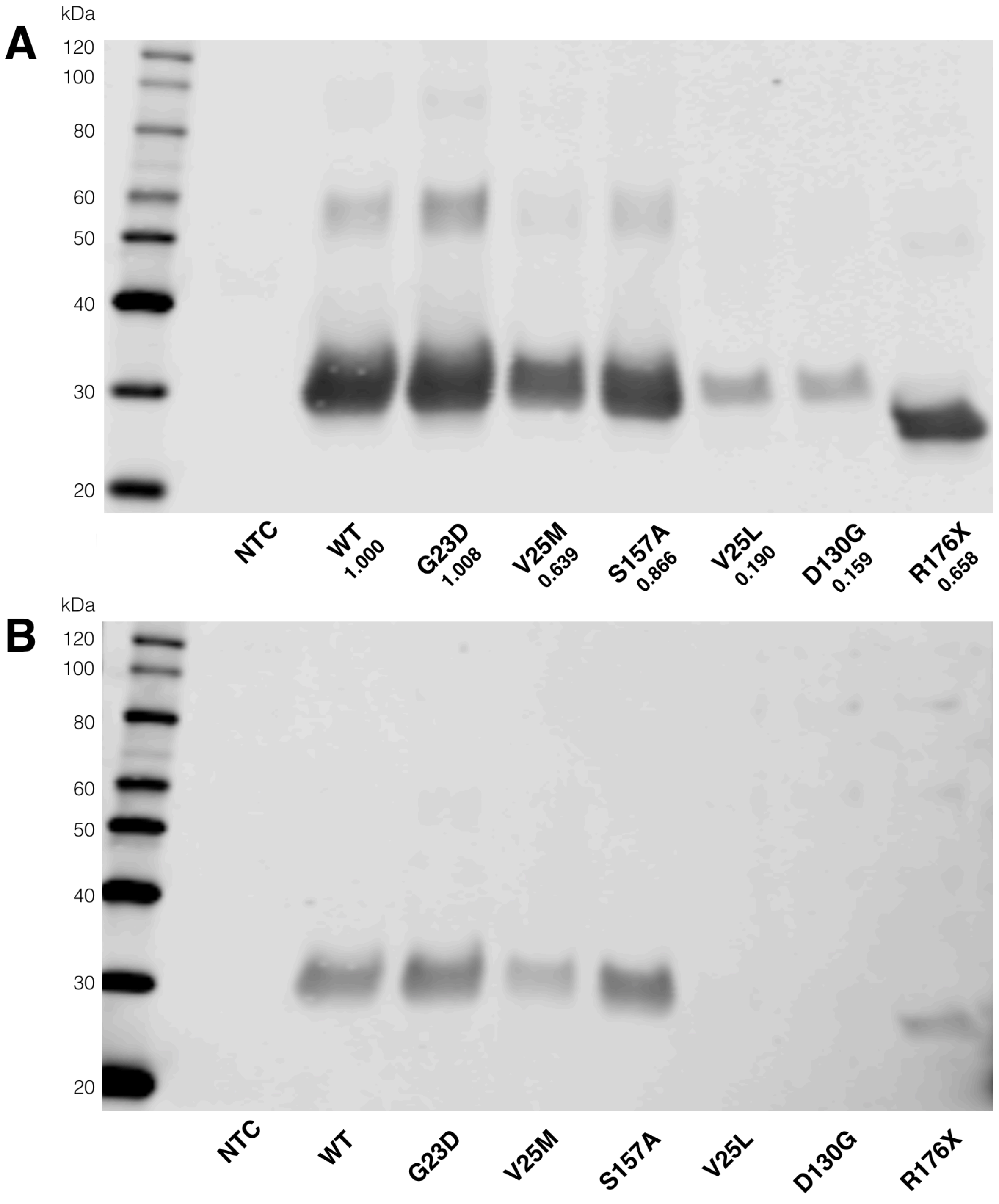

S9 Figure. Western blots of purified RALA proteins. A. Western blot of purified RALA proteins using a RALA antibody, with total amount of protein per lane relative to that used as input for GTPase and GLISA assays. Quantification of the lanes relative to WT is shown. B. Re-probed Western blot of purified RALA proteins from panel A using a 6x-His tag antibody. Note that while similar total levels of protein were loaded for each sample, significantly less V25L and D130G were detected in both blots. NTC, no template control. WT, wild-type.
