# Supplementary material for: De novo mutations in the GTP/GDP-binding region of RALA, a RAS-like small GTPase, cause intellectual disability and developmental delay
Source: PLoS Genet. 2018 Nov 30;14(11):e1007671. doi: 10.1371/journal.pgen.1007671 (PMC6291162; doi:10.1371/journal.pgen.1007671)
Supplement: S10 Fig — (PDF) [file pgen.1007671.s015.pdf]

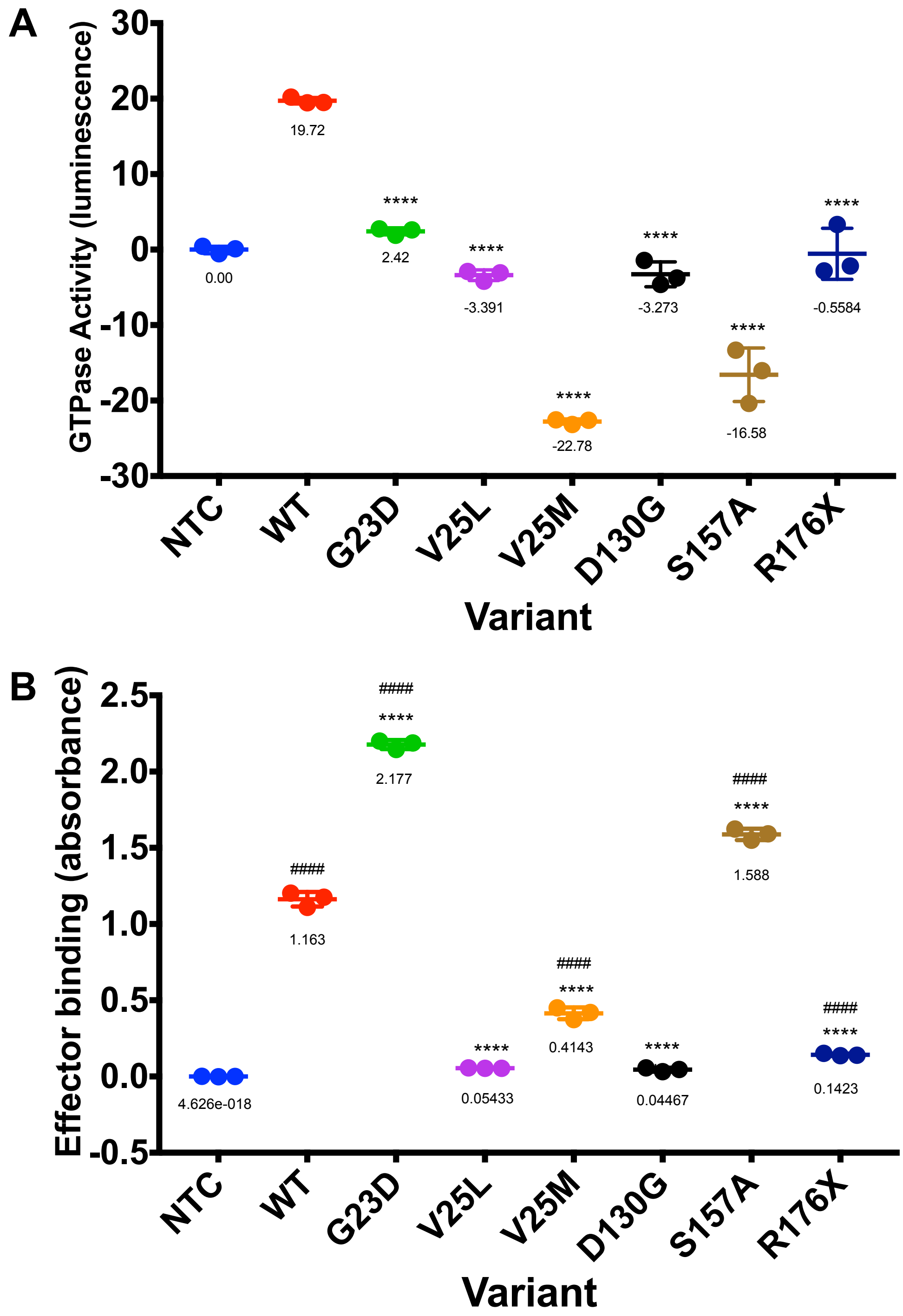

S10 Figure. Results of GTPase activity and RALA effector binding experiments, uncorrected for protein levels. A. GTPase activity of recombinant RALA proteins was assessed using a luminescence assay. Raw luminescence values (measuring remaining free GTP) were subtracted from 100 to calculate activity, and were then normalized to a no template control (NTC). WT, wild-type RALA. **** indicates p-value < 0.0001 compared to WT. Mean values of one experiment performed in triplicate are shown. B. Binding of recombinant RALA proteins to an

effector molecule was assessed using an ELISA assay. Absorbances were normalized to a no template control (NTC). Mean values of one experiment performed in triplicate are shown. WT, wild-type RALA. **** indicates p-value < 0.0001 compared to WT. #### indicates p-value < 0.0001 compared to NTC.
